# HELLS, a chromatin remodeler is highly expressed in pancreatic cancer and downregulation of it impairs tumor growth and sensitizes to cisplatin by reexpressing the tumor suppressor TGFBR3

**DOI:** 10.1002/cam4.3627

**Published:** 2020-12-06

**Authors:** Xuyang Hou, Leping Yang, Kunpeng Wang, Yan Zhou, Qinglong Li, Fanhua Kong, Xi Liu, Jun He

**Affiliations:** ^1^ Department of General Surgery The Second Xiangya Hospital Central South University Changsha Hunan China; ^2^ Department of General Surgery Taizhou Central Hospital Taizhou University Hospital Taizhou Zhejiang China

**Keywords:** chromatin remodeler, cisplatin, epigenetic, HELLS, pancreatic cancer

## Abstract

Pancreatic cancer (PC) is the most malignant cancer type in the digestive system with a poor prognosis. Chemotherapy such as cisplatin is the last chance for PC patients diagnosed with advanced or metastatic disease. Obtaining a deep understanding of the molecular mechanism underlying PC tumorigenesis and identifying optimal biomarkers to estimate chemotherapy sensitivity are essential for PC treatment. The chromatin remodeler HELLS was found to regulate various tumor suppressors through an epigenetic pathway in several cancers. We analyzed HELLS expression in clinical samples by Western blotting and immunohistochemical staining. Next, we identified the variation in tumor growth and cisplatin sensitivity after knockdown of HELLS and explored the downstream mediators of HELLS in PC via RNA‐seq, chromatin immunoprecipitation, and gain‐ and loss‐of‐function assays. We found that HELLS is upregulated in PC tissues and correlates with advanced clinical stage and a poor prognosis, and the knockdown of HELLS leads to tumor growth arrest and increased sensitivity to cisplatin. Mechanistically, the tumor suppressor TGFBR3 is markedly reexpressed after HELLS knockdown; conversely, compromising TGFBR3 rescues HELLS knockdown‐mediated effects in PC cells. Thus, our data provide evidence that HELLS can serve as a potential oncogene and suitable biomarker to evaluate chemotherapy sensitivity via epigenetically silencing the tumor suppressor TGFBR3 in PC.

## Graphical abstract

We found that pancreatic cancer highly expressed a chromatin remodeler, HELLS, and downregulation of it led to decreased growth of pancreatic cancer and improved sensitivity to cisplatin. RNA‐seq, ChIP, and functional assays were used to explore the downstream mechanisms of HELLS and found that the tumor suppressor TGFBR3 was responsible for HELLS knockdown‐mediated effects in pancreatic cancer cells.

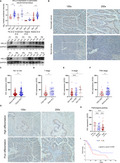

## 1 INTRODUCTION

Pancreatic cancer (PC) is a frequently malignant cancer of the digestive tract, and most patients diagnosed with PC are in the advanced stage with a dismal prognosis. According to GLOBOCAN 2018 estimates, new cases of PC (459,000) almost equaled the number of deaths (432,000) in both males and females worldwide.^1^ In China, the estimated new cases of PC numbered 90.1 per 100,000 people, and the number of deaths was 79.4 per 100,000 in 2015.^2^ PC is poorly responsive to radiation therapy and targeted therapy; hence, combination systemic chemotherapy involving drugs, such as cisplatin, gemcitabine, capecitabine, and oxaliplatin, is the last option for patients with an advanced stage or metastasis.^3^, ^4^ Although an overall survival increase is provided by chemotherapy, the relatively limited patient benefit and predictive markers suggesting individual benefit from chemotherapy are still scarce for PC.^5^ Thus, a better understanding of the molecular mechanisms underlying tumorigenesis and screening for suitable biomarkers for chemotherapy response are essential for PC patients.

Recent advances unveiled the essential role of chromatin remodelers in cancer biology. To disrupt the obstacle of densely packed nucleosomes, chromatin remodelers with the ability of chromatin modification or protein interaction serve to reconstruct nucleosomes, facilitating chromatin accessibility via different mechanisms.^6^, ^7^ At least four families of chromatin remodelers have been identified, including the SWI/SNF family, ISWI family, NuRD family, and INO80 family, in eukaryotes.^8^, ^9^, ^10^, ^11^ Functionally, chromatin remodelers are stimulated by endogenous or exogenous factors and are recruited to specific DNA sites to regulate the transcription of targeted genes, hinting at their potential role in tumorigenesis and drug resistance.^12^ For example, the BRM gene belonging to the SWI/SNF family is commonly suppressed in various cancers by epigenetic silencing and its reexpression leads to impaired tumor growth in vitro and in vivo.^13^ RSF1, a member of the ISWI family, functions as an oncogene in various cancers, correlating with tumor advancement, a worse survival, and a poor prognosis.^14^, ^15^ Recent evidence has also suggested that HELLS, another member of the SWI/SNF family that utilizes energy derived from ATP hydrolysis, is an active chromatin remodeler in tumorigenesis. In nasopharyngeal carcinoma, HELLS upregulation was observed in cancerous tissues to positively correlate with advanced clinical stage and recruitment of the key epigenetic regulator G9a to suppress the activity of fumarate hydratase and drive cancer progression.^16^ Similarly, elevated HELLS expression is intimately associated with malignant clinical stage and poor survival in hepatocellular carcinoma (HCC).^17^ Our previous study demonstrated that HELLS is overexpressed in colorectal cancer and promotes tumor growth.^18^ However, the clinical significance and molecular function of HELLS in PC remains obscure.

Cisplatin is a chemotherapy drug used for PC patients with advanced or metastatic disease, especially for those with BRAC1/2 or PALB2 mutations.^19^ Following the aquation process, the reactive form of cisplatin binds DNA bases to generate DNA adducts and DNA damage.^20^ Mounting evidence has suggested that chromatin remodelers are crucial for the regulation of the DNA damage response in cancer. The ISWI family member SMARCA5 is rapidly recruited to double‐strand breaks (DSBs) in DNA, facilitating the recruitment of RNF168 to regulate DNA repair pathways, including homologous recombination (HR) and nonhomologous end joining (NHEJ).^21^ Interference with SMARCA5 leads to hypersensitivity to DNA damage.^22^ Similarly, SWI/SNF family members, such as BRM and BRG1, are efficiently localized to DSB sites induced by doxorubicin and cisplatin in an ATM‐dependent manner, increasing damage signaling and promoting chromatin relaxation.^23^ Notably, current evidence indicates a highly context‐dependent pattern of chromatin remodelers in the DNA damage response.^21^ A similar observation was also found in HELLS. For example, the knockdown of HELLS decreased γH2AX, a DNA damage marker after cisplatin treatment in nasopharyngeal carcinoma cells, whereas the deletion of HELLS delayed the accumulation of NHEJ components and increased γH2AX signals in DNA damage sites, resulting in increased apoptosis in HEK293 cells.^24^, ^25^ Thus, the linkage between HELLS and the DNA damage response or cell death is highly controversial in distinct contexts, and its exact role in PC exerted by cisplatin treatment needs detailed investigation.

In this study, we first analyzed the expression of HELLS in clinical samples and retrieved the mRNA profile of PC from the online database to integrate HELLS expression with clinicopathological features and prognosis. The knockdown of HELLS significantly impaired tumor growth in vitro and in vivo and increased the sensitivity to cisplatin treatment in PC. Moreover, tumor suppressor TGFBR3 was significantly reexpressed upon HELLS knockdown and served as a downstream mediator of HELLS; knockdown of TGFBR3 largely rescued HELLS knockdown‐mediated effects on PC cells. Thus, we found that HELLS determined the growth and chemotherapy sensitivity of PC possibly through epigenetic silencing of TGFBR3.

## 2 MATERIALS AND METHODS

### 2.1 Human samples and PC cell lines

This study was approved by the Ethics Committee for Human Research, Central South University and was conducted according to the approved guidelines. The patients whose tissues were used provided written informed consent in accordance with the Declaration of Helsinki. For fresh samples, 12 patients diagnosed with PC were included and the cancerous and paracancerous tissues were collected after surgical resection. Fresh samples were preserved at −80°C. Additionally, another cohort of 87 pairs of PC samples was collected for immunohistochemical staining. These samples were maintained in paraffin packaging for prolonged preservation. The PC cell lines Panc‐1, BxPC‐3, and CFPAC‐1 were purchased from ZSBIO, China, which also performs cell line STR genotyping. For cell culture, Panc‐1 and BxPC‐3 cells were cultured in DMEM medium supplemented with 10% of fetal bovine serum (FBS) and 1% of penicillin‐streptomycin, while CFPAC‐1 cells were maintained in RPMI‐1640 medium supplemented with 10% of FBS and 1% of penicillin‐streptomycin. All the cells were placed in a humidified incubator with 5% of CO_2_ at 37°C.

### 2.2 Cell viability/proliferation assay

The cell viability/proliferation rate was tested using the CCK‐8 kit (GeneView). After transfection, the cells were plated in 96‐well plates with at least three replicates per group and were cultured for 24 hours at 37°C. CCK‐8 reagents were added to each well and cultured for 3 hours, followed by determination of the OD values at 450 nm utilizing a microplate spectrophotometer (Thermo Fisher). All the values were normalized to the control group without adding cells and were presented as means ±SD.

### 2.3 Colony formation assay

After transfection, the PC cells were diluted through a dilution gradient, and then, 5,000 cells were seeded per well in 6‐well plates. The cells were cultured for 7 days, followed by washing with PBS two times, fixing with 4% of paraformaldehyde, and staining with 1% of crystal violet staining solution (Beyotime, China). Images of colonies were captured by a digital camera.

### 2.4 Subcutaneous xenograft model

BALB/c nude mice received human care in compliance with the guidelines implemented at Second Xiangya Hospital, Central South University. The study was performed according to the international, national, and institutional rules considering animal experiments and biodiversity rights. Briefly, 3 × 10^6^ Panc‐1 cells transfected with HELLS siRNA or control siRNA were subcutaneously injected into the right dorsal of 6‐week‐old male nude mice (n = 5). After feeding for 4 weeks, the mice were sacrificed and tumors were collected.

### 2.5 Western blot analysis

PC samples and cells were lysed in RIPA buffer supplemented by protease and phosphatase inhibitors (TargetMol) and incubated on ice for 30 minutes. The supernatant was collected after discarding the sedimentation. The denatured proteins were added to the chamber for SDS‐PAGE, followed by electrotransfer onto PVDF. After blocking with 3% of BSA for 1 hours, the membranes were incubated with diluted primary antibody overnight at 4°C. On the following day, the primary antibody was discarded and the membranes were washed three times with TBST, followed by incubation with diluted secondary antibody for 1 hours at room temperature. The immune complexes were detected via an enhanced chemiluminescence system (Life Tec). Analysis and quantification of the bands were performed using ImageJ software (Version 11). The primary antibodies involved in this work included the following: HELLS (1:1000; Abclonal), TGFBR3 (1:1000; Abclonal), γH2AX (1:1000; Abclonal), caspase 3/cleaved caspase 3 (1:1000; CST), caspase 9/cleaved caspase 9 (1:1000; CST), Bax (1:1000; Abclonal), and GAPDH (1:1000; Abclonal). Secondary antibodies were purchased from Abclonal, China.

### 2.6 Quantitative real‐time PCR (qRT‐PCR)

The RNA of tissues and cells was extracted using standard methods, as previously described.^26^ Briefly, TRIzol reagent was added to the samples, followed by lysis for 10 minutes, centrifugation at 12000 g for 15 minutes, and then, reduction with isopropanol. The RNA purity and concentration were tested using a NanoDrop 2000 spectrophotometer (Thermo Fisher). cDNA was synthesized using a high‐capacity cDNA reverse transcription kit (Life Tec) according to the manufacturer's guidelines. The primers were listed as follows: HELLS, 5′‐TAGAGAGTCGACAGAAATTCGG‐3′ (forward) and 5′‐CCTCATAACTGGCTTCTCTTCA‐3′ (reverse); TGFBR3, 5′‐GGAGATATGGATGAAGGAGAT‐3′ (forward) and 5′‐GCAGTGAGGTGTTGAAGA‐3′ (reverse); TGFBR3 R1, 5′‐CATCAGAGCGTGACAACA‐3′ (forward) and 5′‐TCATTTGGTTCCTTGGTCTT‐3′ (reverse); TGFBR3 R2, 5′‐TGAGAAGCGGAGGTTGTA‐3′ (forward) and 5′‐GCGATATGAACGACAGTCT‐3′ (reverse); TGFBR3 R3, 5′‐AATGGTTCAGTGAGGAGATT‐3′ (forward) and 5′‐GAGGAGGCTTCTTATGACAT‐3′ (reverse); TGFBR3 R4, 5′‐GAACTCCTGACCTCAAGTG‐3′ (forward) and 5′‐TTGCTCCTCCTTACCTTCT‐3′ (reverse); TGFBR3 R5, 5′‐TGTTGATGGTTACTGTTGTG‐3′ (forward) and 5′‐ GGCTGAGGCAAGAGAATC‐3′ (reverse). 2× Universal SYBR Green Fast qPCR Mix (Abclonal, China) was used for qRT‐PCR in a LightCycler 96 system (Roche).

### 2.7 Immunohistochemical staining (IHC)

The IHC analyses of clinical samples were generally performed as previously described.^26^ Briefly, 4‐μM‐thick sections were prepared from paraffin‐embedded samples, which were then deparaffinized sequentially, followed by incubation with 3% of H_2_O_2_ in the dark for 15 minutes, and heat‐induced epitope retrieval using sodium citrate buffer (10 mM sodium citrate and 0.05% Tween 20 at pH 6.0) at 96°C for 30 minutes. After washing with PBS three times, the sections were incubated with rabbit antihuman HELLS or antihuman cleaved caspase‐3 primary antibody for 2 hours. Rabbit control IgG served as the control antibody. After incubation of Solution A (ChemMateTMEnVision+/HRP) for 30 minutes, DAB staining and hematoxylin counterstaining were performed. The sections were then dehydrated, soaked in xylene, and mounted with neutral balsam.

### 2.8 Immunofluorescence

The cells were seeded in 24‐well plates and cultured for 24 hours, and then, were washed twice with PBS and fixed with 4% of paraformaldehyde and permeabilized with 0.25% of Triton X‐100 (Sigma‐ALDRICH). Next, 3% of bovine serum albumin was used to block nonspecific antigen‐antibody reaction. After incubation of primary anti‐γH2AX antibody overnight and washing with PBST three times, the cells were incubated with donkey anti‐rabbit Alexa Fluor 488 (Invitrogen) for 1 hour at room temperature. The cells were rinsed and stained with DAPI (Invitrogen). The immunofluorescence signal was detected using a fluorescence microscope (Olympus Inc.).

### 2.9 EdU assay

Cells were seeded in 24‐well plates and cultured for 24 hours. On the following day, the cells were rinsed once with PBS, incubated with EdU in culture medium for 2 hours at 37°C, and then, rinsed three times with PBS. After permeabilization with 0.25% of Triton X‐100 (Sigma‐Aldrich), the cells were stained with DAPI and captured using a fluorescence microscope (Olympus Inc.).

#### 2.9.1 siRNA transfection

For transfection, the cells were seeded in 6‐well plates and cultured for 24 hours at 50% confluence. Next, siRNAs or negative controls were transfected using Lipofectamine™ RNAiMAX transfection reagent (Invitrogen). The targeted sequences of HELLS were GCTCGCATGTCTTGGGATA, GCAGCAGATACAGTTATCA and for TGFBR3 were GGAGATGCTTCCCTGTTCA and GGGCCATGATGCAGAATAA. After an incubation period of 48 hours, the cells were prepared for functional assays.

#### 2.9.2 Flow cytometry assay

The percentage of apoptosis was detected by flow cytometry after cells were stained with Annexin V and PI. The Annexin V/PI Apoptosis Detection Kit was purchased from Yeasen, China and used according to the manufacturer's guidelines. Briefly, cells in various groups were harvested, washed three times with PBS, resuspended in 1× binding buffer and stained with the Annexin V and PI solution for 30 minutes at 37°C without light. Next, the cells were sent for testing by BD FACSARIA II flow cytometry (Becton Dickinson).

#### 2.9.3 RNA‐sequencing (RNA‐seq)

A total amount of 1 µg RNA per sample was used as input material for the RNA sample preparations. The sequencing libraries were generated using the NEBNext^®^ UltraTM RNA Library Prep Kit. After cluster generation, the library preparations were sequenced using an Illumina NovaSeq platform and 150 bp paired‐end reads were generated. The RNA‐sequence procedure and data analysis were performed by Novogene, China.

#### 2.9.4 Cromatin immunoprecipitation (ChIP) assay

ChIP assay was performed using a ChIP kit (Abcam) according to the manufacturer's recommendations. Briefly, cells were seeded at 5 × 10^6^ cells in a 10‐cm dish and cultured for 24 hours. On the following day, the cells were fixed in formaldehyde for 10 min and stopped by glycine. Next, the cells were washed twice with PBS and harvested on ice. The cell pellets were sonicated under the proper conditions. ChIP buffer/PI mix was then added to the sheared chromatin, followed by the addition of the primary antibody and incubation overnight at 4°C and preparation of the antibody binding beads. The antibody/chromatin samples were collected by centrifugation, and the supernatant was removed. After purification by proteinase K, the DNA slurry was harvested by centrifugation. The purified DNA after ChIP was analyzed by qRT‐PCR using Universal SYBR Green Fast qPCR mix (Abclonal). The ChIP grade antibody was purchased from CST, and the primers are listed in qRT‐PCR section.

#### 2.9.5 Statistical analysis

All statistical analyses were performed using Prism software (GraphPad Prism 6). Two‐tailed Student's *t*‐test was used to assess significant differences between two groups. For three or more groups, one‐way ANOVA was used. The threshold of *p*‐values was 0.05, and values less than 0.05 were considered statistically significant.

## 3 RESULTS

### 3.1 HELLS is upregulated in PC and correlates with TNM stage and prognosis

**FIGURE 1:**
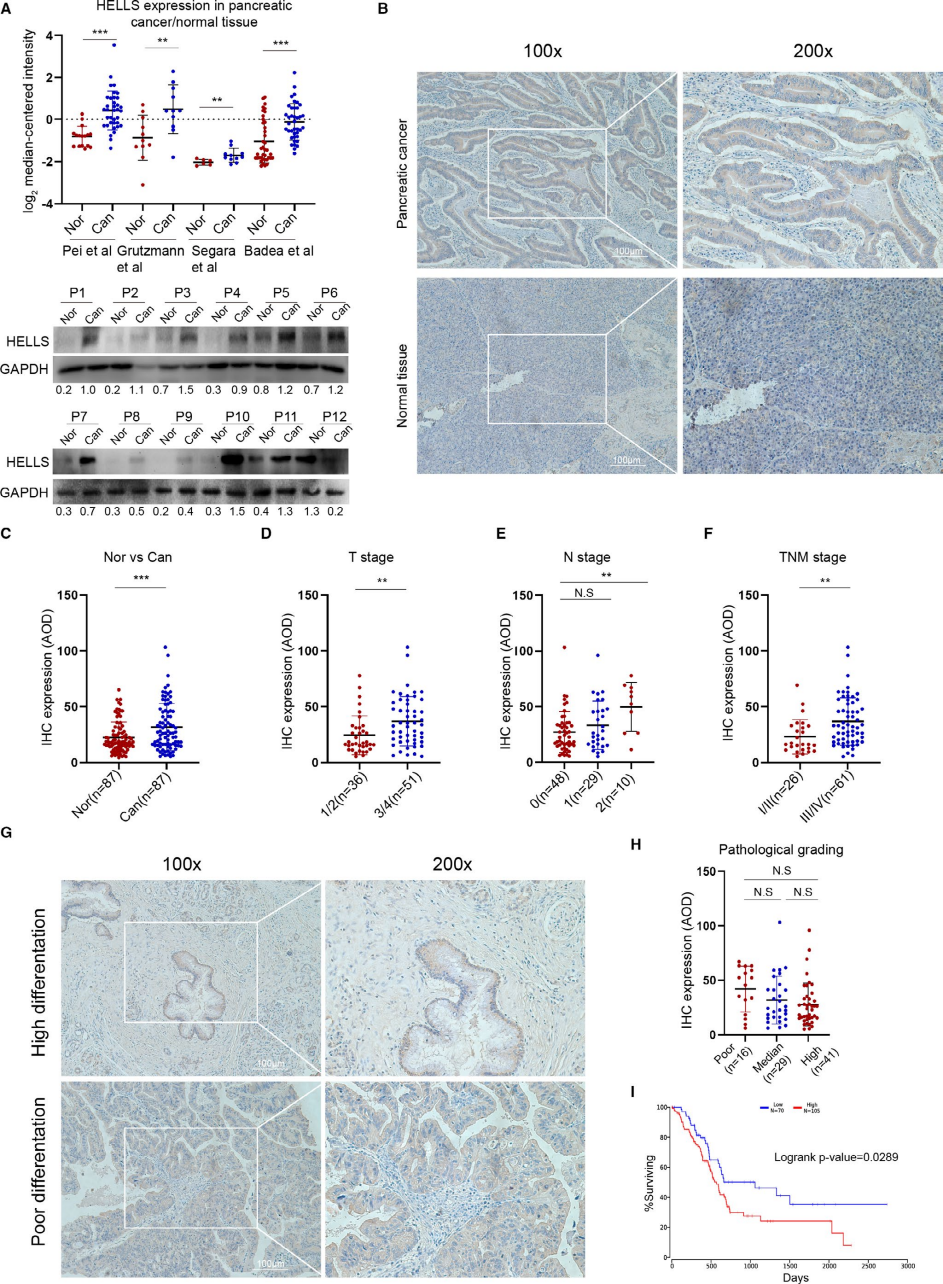
HELLS is upregulated in PC and correlates with clinical parameters. (A) Four data sets from the Oncomine database (www.oncomine.org) were selected to compare HELLS mRNA expression between PC tissues and normal tissues. Twelve pairs of fresh samples from PC patients were collected, and the protein expression of HELLS was tested by Western blotting. The numbers blow the bands indicate the expression of HELLS relative to GAPDH in each sample. Nor, normal; Can, cancer; P, patient. (B) IHC was performed to detect HELLS expression in a cohort of 87 PC patients. Representative images of HELLS staining in PC tissues and paracancerous tissues are shown. (C) The IHC images were analyzed by ImageJ and quantified by assessing the average optical density (AOD) in each sample. The scatter plot shows the AOD of each paracancerous and corresponding cancerous tissue. The means of AOD in paracancerous and cancerous tissues were 22.33 and 31.72, respectively. (D) The scatter plot shows HELLS staining stratified by T stage; n(T1/2) = 36, n(T3/4) = 51. (E) The scatter plot shows HELLS staining stratified by N stage; n(N0) = 48, n(N1) = 29, n(N2) = 10. (F) The scatter plot shows HELLS staining stratified by TNM stage; n(TNM I/II) = 26, n(TNM III/IV) = 61. (G) Representative images of highly differentiated and poorly differentiated PC tissues. (H) The scatter plot shows HELLS staining stratified by pathological grading and includes poorly differentiated (n = 16), median differentiated (n = 29), and highly differentiated grades (n = 41). (I) Survival probability of PC patients with high (n = 105) or low (n = 70) HELLS mRNA expression. The data were derived from the TCGA PAAD program. N.S, no significance; ** *p* < 0.01, ***p < 0.001

To examine the expression of HELLS between pancreatic cancerous and normal tissues, we first queried the data from the Oncomine database (www.oncomine.org). Four data sets were included and showed significantly enhanced expression of HELLS mRNA in cancerous tissues compared with normal tissues (Figure 1A upper). In fresh samples collected from surgical resection, HELLS protein was upregulated in most of the cancerous tissues compared with the corresponding paracancerous ones (Figure 1A lower). We further determined HELLS expression in a cohort of 87 patients using the IHC assay, and representative staining is shown in Figure 1B. The results clearly showed higher expression of HELLS in cancerous tissues than in paracancerous ones (Figure 1C).

To determine the clinical significance of HELLS in PC, we correlated its expression with the clinical parameters. High expression of HELLS was correlated with high grades of T stage, N stage, and TNM clinical stage (Figure 1D–F), but no significant association was observed with M stage, vascular invasion, and patient gender and age (data were not shown). HELLS expression was also not correlated with pathological grading (Figure 1G,H). To investigate the prognostic value of HELLS, data from the PAAD program was extracted and reanalyzed using the OncoLnc online tool (www.oncolnc.org). High expression of HELLS indicated a poor prognosis for PC patients. Collectively, these data demonstrated that PC upregulates the expression of HELLS and is correlated with T stage, N stage, TNM clinical stage, and prognosis.

### 3.2 Downregulation of HELLS impairs PC growth in vitro and in vivo

**FIGURE 2:**
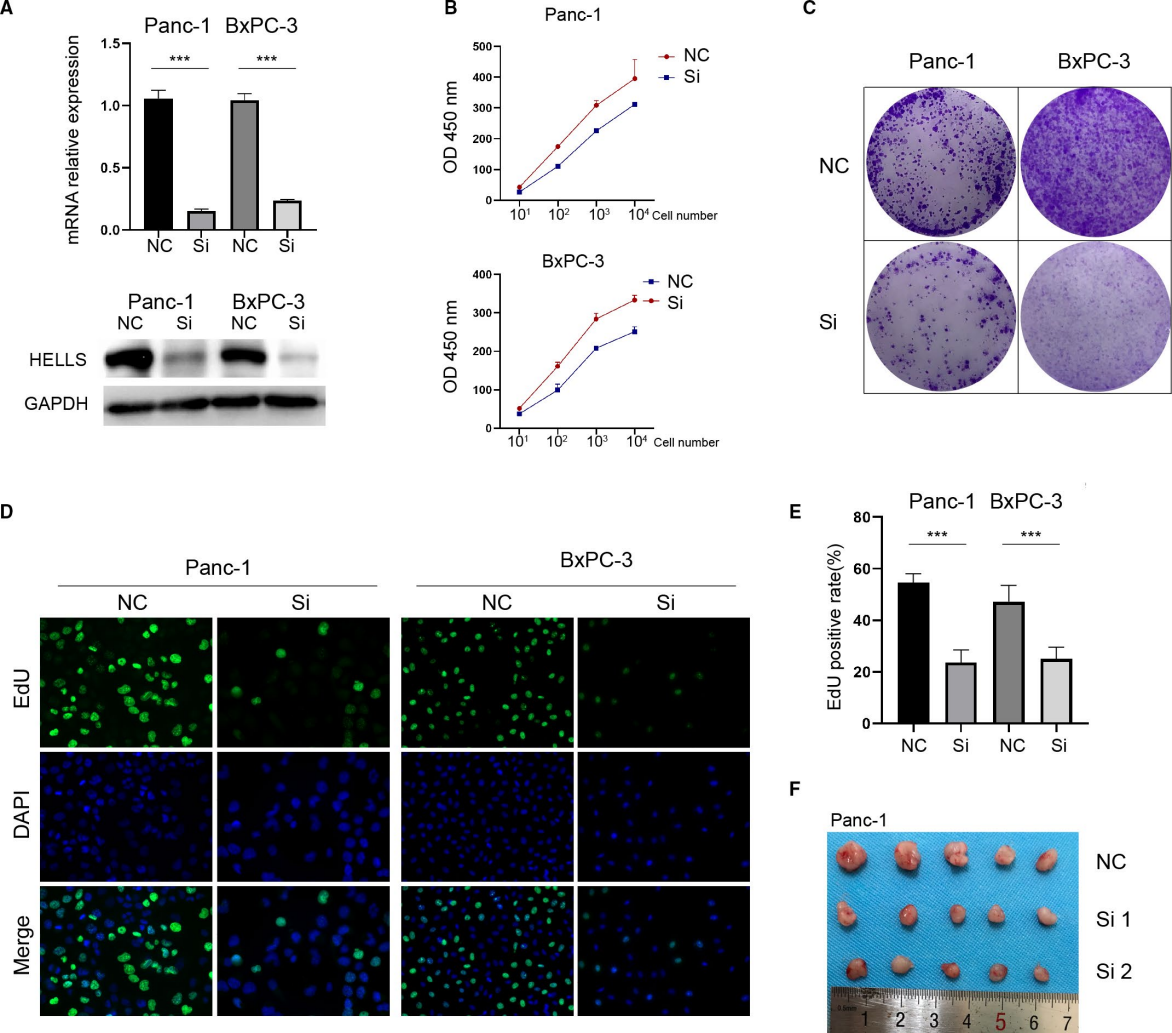
Downregulation of HELLS impairs PC growth in vitro and in vivo. (A) siRNA was used to inhibit KDM1A or KDM3A expression in Panc‐1 and CFPAC‐1 cells and was verified by qRT‐PCR (left) and Western blotting (right). (B) PC cells were seeded at 10, 10^2^, 10^3^, and 10^4^ per well, and CCK‐8 was used to detect cell numbers after 48 hours. Knockdown of HELLS reduces cell proliferation. (C) The colony formation assay was performed to determine the ability of colony formation. Knockdown of HELLS reduced colony formation in Panc‐1 and BxPC‐3 cells. (D–E) The EdU assay showed that the knockdown of HELLS reduces cell proliferation in Panc‐1 and BxPC‐3 cells. (F) Panc‐1 cells transfected by siRNA or control were subcutaneously injected into nude mice. After 4 weeks, the mice were sacrificed and tumors were collected. NC, negative control; Si, siRNA; ***p < 0.001

Our previous work found that the inhibition of HELLS leads to cell cycle arrest in colorectal cancer. Thus, we wanted to know whether HELLS expression could determine the growth of PC. siRNA successfully downregulated HELLS expression in two PC cell lines, Panc‐1 and BxPC‐3, as verified by mRNA and protein examination (Figure 2A). Next, the CCK‐8 and colony formation assays were performed. Knockdown of HELLS significantly impaired the cell proliferation rate and reduced the ability of colony formation (Figure 2B,C). Additionally, the EdU assay confirmed that the knockdown of HELLS reduced cell proliferation (Figure 2D,E). The data indicated that the downregulation of HELLS hampers PC growth in vitro. To further verify the role of HELLS in vivo, Panc‐1 cells were subcutaneously injected into nude mice after siRNA treatment (two strands of siRNA were used). Compared with the control group, the tumor volume of the siRNA group was significantly reduced (Figure 2F). Thus, these data collectively indicated that the downregulation of HELLS impairs PC growth in vitro and in vivo.

### 3.3 Downregulation of HELLS sensitizes PC to cisplatin treatment via enhanced DNA damage and apoptosis

**FIGURE 3:**
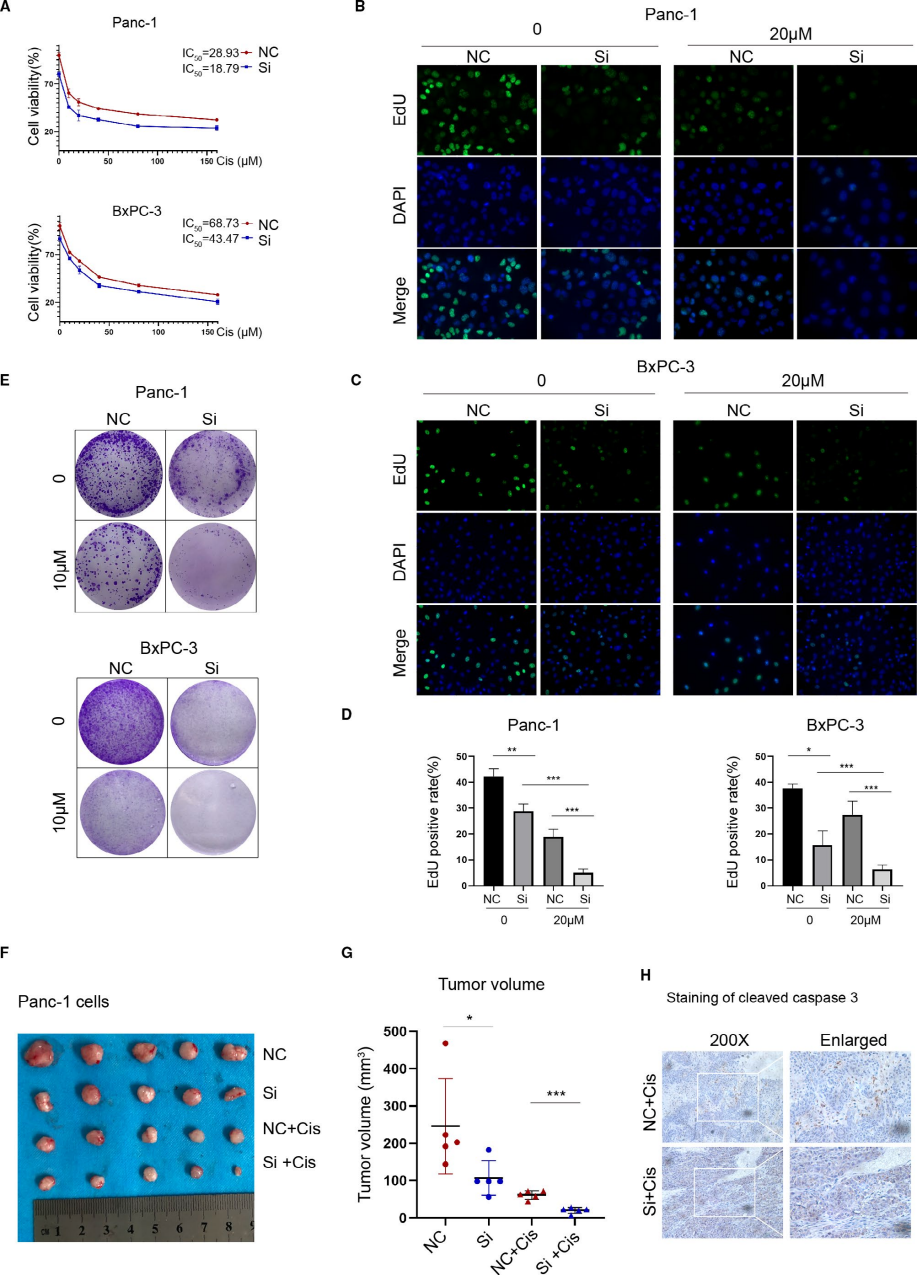
Downregulation of HELLS sensitizes PC to cisplatin. (A) Cell viability was detected by the CCK‐8 assay in PC cells treated with various concentrations of cisplatin for 24 hours. The IC_50_ values of the NC and siRNA groups were 28.93 and 18.79 μM, respectively, in Panc‐1 cells, and 68.73 and 43.47 μM, respectively, in BxPC‐3 cells. (B–D) Knockdown of HELLS reduced the cell proliferation of Panc‐1 and BxPC‐3 cells treated with 20 μM cisplatin. (E) A total of 5,000 cells per well was seeded and cultured for 4 days, followed by treatment with cisplatin and culture for another 3 days. Knockdown of HELLS remarkably inhibits the colony formation of Panc‐1 and BxPC‐3 cells treated with 10 μM cisplatin. (F) Panc‐1 cells transfected by siRNA or control were subcutaneously injected into nude mice. From week 1, NC+Cis and Si+Cis groups were treated with intraperitoneal injection of cisplatin (3 mg/kg) every 3 day for seven times. (G) Three days after the last treatment, the animals were sacrificed and the tumors were collected for volume measurement and IHC analysis. (H) IHC staining of cleaved caspase‐3 in NC+Cis and Si+Cis groups. Cis, cisplatin; NC, negative control; Si, siRNA; *p < 0.05, **p < 0.01, ***p < 0.001

Because platinum drugs were preferred for the chemotherapy of PC, we tested whether the HELLS expression level could determine the sensitization to PC cells to cisplatin. Panc‐1 and BxPC‐3 cells with or without HELLS siRNA were treated with various concentrations of cisplatin for 24 hours, and cell viability was detected by the CCK‐8 assay. The half‐maximal inhibitory concentration (IC_50_) of HELLS siRNA was markedly decreased in both cell lines (18.79 vs. 28.93 μM in Panc‐1 cells; 43.47 vs. 68.73 μM in BxPC‐3 cells; Figure 3A). Accordingly, the EdU assay indicated that the cell proliferation rate was also significantly reduced in the HELLS siRNA group in the presence of cisplatin in both cells (Figure 3B–D). Furthermore, HELLS siRNA remarkably reduced colony formation ability when treated with cisplatin in both cells (Figure 3E). In vivo assay was also conducted. Compared to control group, HELLS siRNA markedly improved the sensitivity of PC to cisplatin reflected by smaller tumor volume and enhanced cleaved caspase‐3 staining (Figure 3F–H). These data demonstrated the downregulation of HELLS sensitizes PC to cisplatin.

**FIGURE 4:**
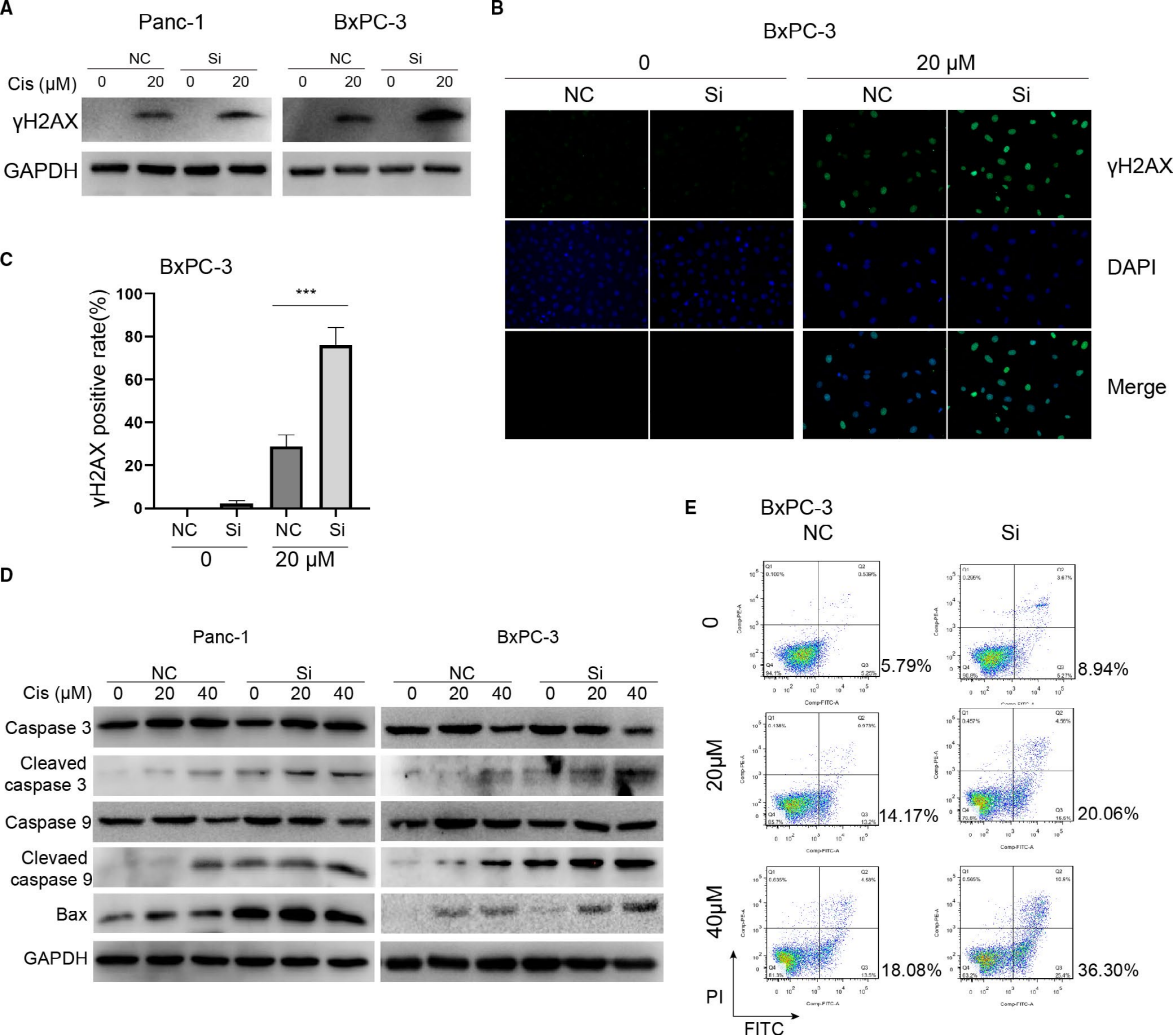
Downregulation of HELLS promotes DNA damage and apoptosis induced by cisplatin. (A–C) γh2AX served as a sensitive marker for DNA damage and was detected by Western blot and immunofluorescence. The knockdown of HELLS led to enhanced expression of γh2AX in Panc‐1 and BxPC‐3 cells. (D) Detection and comparison of the expression of caspase 3, cleaved caspase 3, caspase 9, cleaved caspase 9 and Bax between the HELLS siRNA group and control group treated with 0, 20, or 40 μM cisplatin. These proapoptosis proteins were consistently upregulated in the HELLS siRNA group compared with those in the control group, indicating that the knockdown of HELLS facilitates apoptosis induced by cisplatin. (E) The proportion of apoptosis was quantitatively examined by flow cytometry. The apoptosis rates were 20.06% and 36.30% when treated with 20 and 40 μM cisplatin in the siRNA group versus 14.17% and 18.08% in the control group, respectively. Cis, cisplatin; NC, negative control; Si, siRNA; ***p < 0.001

Cisplatin is rapidly aquated, acquiring high affinity to DNA and leading to DNA damage and activation of the DNA damage response. We tested whether the downregulation of HELLS causes enhanced DNA damage in PC cells. γh2AX, the phosphorylated form of H2AX that correlates well with double‐strand breaks (DSB) of DNA, served as the most sensitive marker for DNA damage. The downregulation of HELLS did not yield enhanced DNA damage in the absence of cisplatin. However, in the presence of cisplatin, HELLS siRNA significantly accelerated DNA damage in both cells (Figure 4A–C). The mitochondrial cell death pathway was induced by cisplatin in a dose‐dependent pattern, as reflected by the increased expression of cleaved caspase 3, cleaved caspase 9, and Bax (Figure 4D). At the same concentration of cisplatin, HELLS siRNA led to increased expression of proapoptotic proteins compared with that in the control group in both cell lines. Accordingly, the proportion of apoptosis was quantitated by flow cytometry and the same trend was observed (36.3% vs. 18.08% in the HELLS siRNA and control group with 40 μM cisplatin, respectively; Figure 4E). Thus, these data demonstrated that the inhibition of HELLS sensitizes PC cells to cisplatin by elevating DNA damage and apoptosis.

### 3.4 HELLS silences tumor suppressor TGFBR3 through an epigenetic pathway in PC cells

**FIGURE 5:**
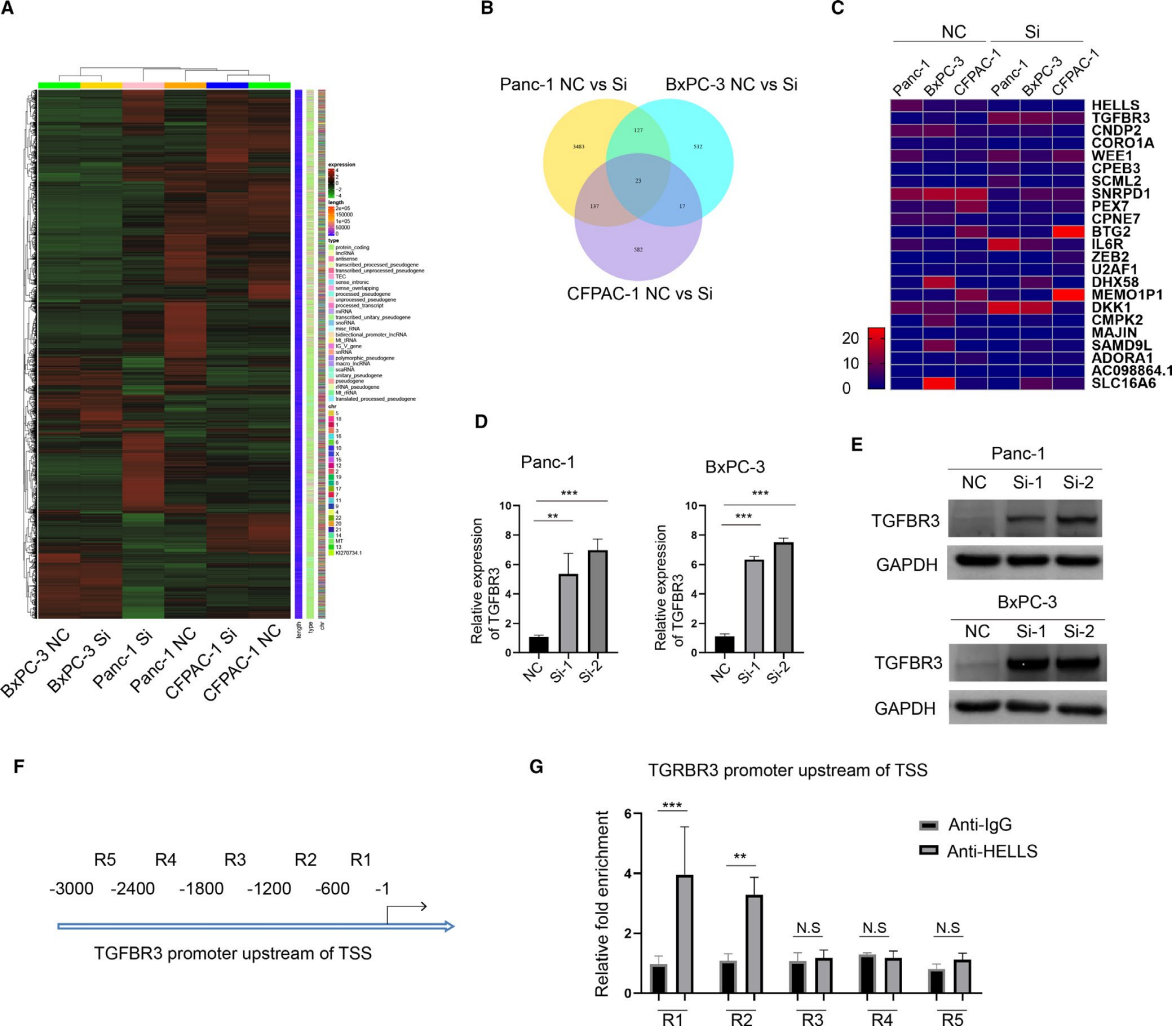
HELLS suppresses the tumor suppressor TGFBR3 via epigenetic regulation in PC cells. (A) RNA‐seq was performed to compare whole‐gene expression variations between the HELLS siRNA group and control group in Panc‐1, BxPC‐3, and CFPAC‐1 cells. (B) In total, 3770 genes, 699 genes and 759 genes were screened at the absolute value of fold change >2 in Panc‐1, BxPC‐3, and CFPAC‐1 cells, respectively. Only 23 genes consistently differed among the three cell lines. (C) Redraw of the heatmap of differentiated genes among the three cell lines. (D) TGFBR3 mRNA is reexpressed by the knockdown of HELLS in Panc‐1 and BxPC‐3 cells. (E) TGFBR3 protein is reexpressed by the knockdown of HELLS in Panc‐1 and BxPC‐3 cells. (F) Schematic division of the TGFBR3 promoter upstream of the TSS. Primers for each division were designed for qRT‐PCR. (G) ChIP assay was performed to detect DNA bonding with HELLS. qRT‐PCR with TGFBR3 primers was performed and semiquantitatively analyzed. Compared with the other primers, significant fold enrichment of R1 and R2 products was observed between the anti‐IgG group and anti‐HELLS group. Cis, cisplatin; N.S., no significance; ** *p* < 0.01, ***p < 0.001

Previous reports have demonstrated that HELLS increases the occupancy of nucleosomes, which block the accessibility of enhancers and hampers gene transcription in HCC; however, HELLS function in PC was unclear. To delineate the downstream function of HELLS in PC, we analyzed global gene expression variations by RNA‐seq in three PC cell lines—Panc‐1, BxPC‐3, and CFPAC‐1—after HELLS knockdown (Figure 5A). Among them, with a threshold of fold change >2 or <−2, 3,770 genes in Panc‐1 cells, 699 genes in BxPC‐3 cells, and 759 genes in CFPAC‐1 cell were identified and 23 genes commonly differed among the three cell lines (Figure 5B,C). We focused on TGFBR3, which is a well characterized tumor suppressor in several tumors and was highly reexpressed in the HELLS siRNA group. The RNA‐seq data were verified by qRT‐PCR in Panc‐1 and BxPC‐3 cells in which HELLS were downregulated by two distinct siRNAs (Figure 5D). Furthermore, TGFBR3 protein was much enhanced, as determined by Western blotting in HELLS siRNA cells (Figure 5E).

HELLS hinders the nucleosome‐free region (NFR) at the transcription start site (TSS) of targeted genes. Thus, we tested whether HELLS binds to the promoter regions of TGFBR3 by the ChIP assay. After purification, DNA was used for qPCR to determine the relative fold enrichment between the anti‐HELLS group and IgG group. Figure 5F shows the schematic division of the 3000‐bp region upstream of TGFBR3 TSS. In accordance with a previous report that HELLS can bind to the promoter region of targeted genes, in on our examination, HELLS targeted the TGFBR3 promoter region of R1 and R2 in PC cells (Figure 5G). Collectively, these data indicated that HELLS epigenetically silences the tumor suppressor TGFBR3 in PC cells, and the function of HELLS/TGFBR3 axis remains to be elucidated.

### 3.5 Knockdown of TGFBR3 rescues HELLS knockdown‐mediated effects in PC cells

**FIGURE 6:**
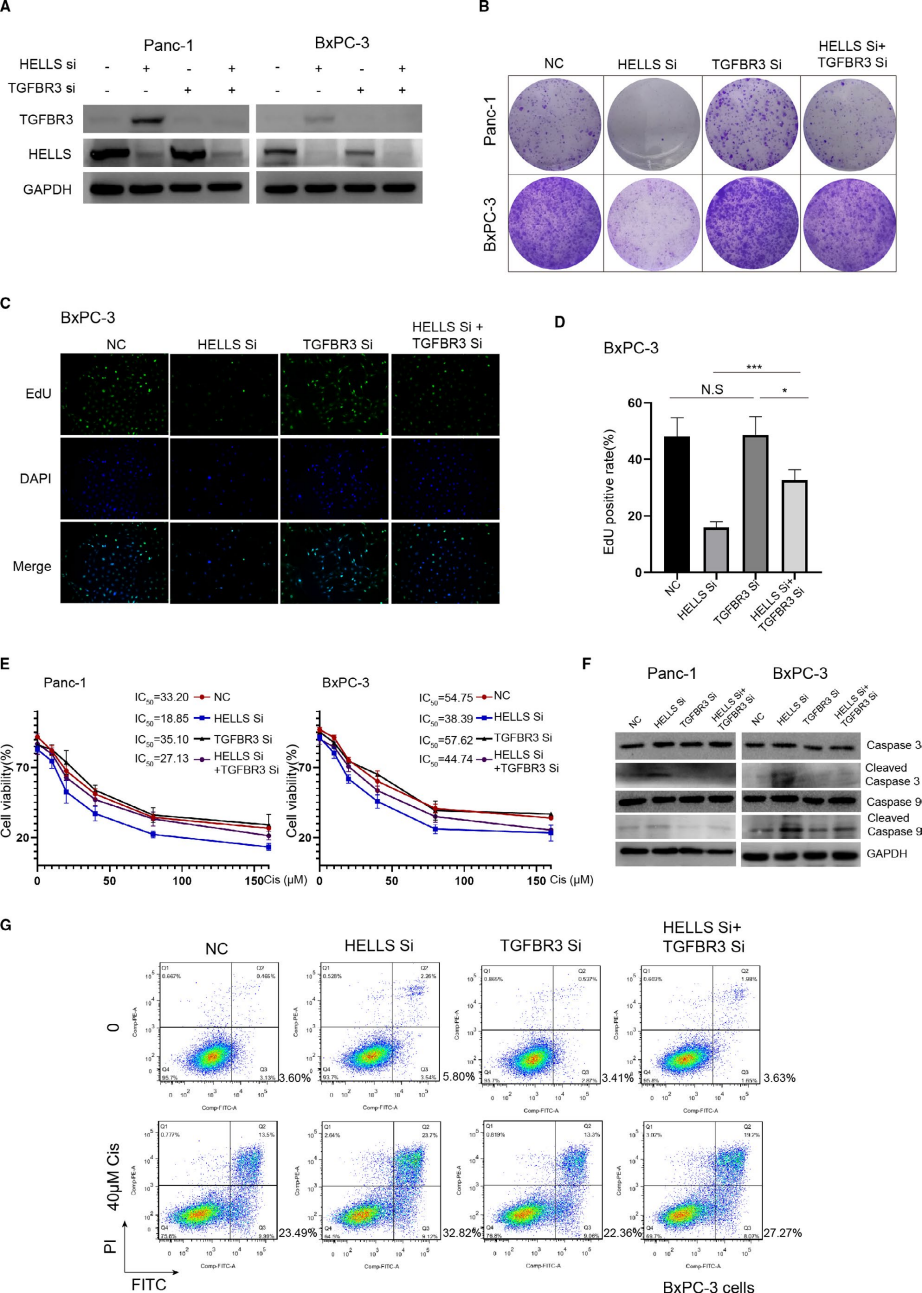
Knockdown of TGFBR3 rescues HELLS knockdown‐mediated effects in PC cells. (A) TGFBR3 siRNA abrogates the increased expression of TGFBR3 by HELLS siRNA in Panc‐1 and BxPC‐3 cells. (B) TGFBR3 siRNA slightly improves the ability of colony formation, and rescues the inhibitory effect of HELLS siRNA in Panc‐1 and BxPC‐3 cells. (C–D) In BxPC‐3 cells, knockdown of TGFBR3 does not influence cell proliferation but significantly improves it when HELLS was downregulated. (E) Knockdown of TGFBR3 does not affect the sensitivity to cisplatin in Panc‐1 and BxPC‐3 cells but increases the cell viability in cells transfected with HELLS siRNA. (F) Knockdown of TGFBR3 reduces HELLS‐mediated improvement of cleaved caspase‐3 and cleaved caspase‐9. (G) Quantitatively, knockdown of TGFBR3 inhibits apoptosis in BxPC‐3 cells transfected with HELLS siRNA and treated with 40 μM cisplatin. Cis, cisplatin; NC, negative control; Si, siRNA; N.S., no significance; *p < 0.05, ***p < 0.001

To explore the role of TGFBR3 in the HELLS knockdown‐mediated effect in PC cells, gain‐ and loss‐of‐function assays were performed. We used siRNA to knockdown TGFBR3 in PC cells. Western blotting showed that TGFBR3 siRNA markedly inhibits TGFBR3 protein regardless of the HELLS status (Figure 6A). The colony formation assay indicated that TGFBR3 siRNA improves the ability of colony formation by partially reversing HELLS knockdown‐mediated growth arrest in Panc‐1 and BxPC‐3 cells (Figure 6B). A similar result was obtained from the EdU assay in both cell lines (Figure 6C,D). These data suggested that TGFBR3 is an essential downstream mediator of HELLS and its knockdown can rescue HELLS knockdown‐mediated growth arrest.

Next, we tested whether the knockdown of TGFBR3 also reverses HELLS knockdown‐mediated sensitization to cisplatin. The CCK‐8 assay was used to detect the cell viability, and the IC_50_ values of the control group, HELLS siRNA group, TGFBR3 siRNA group, and HELLS siRNA+TGFBR3 siRNA group were 33.20, 18.85, 35.10, and 27.13 μM, respectively, in Panc‐1 cells; the results with BxPC‐3 cells were well correlated with those of Panc‐1 cells (Figure 6E). We next detected the expression of cleaved caspase‐3 and cleaved caspase‐9 in each group. TGFBR3 siRNA attenuated cisplatin‐induced mitochondrial cell death in the presence of HELLS knockdown (Figure 6F). Quantitatively, the proportion of apoptosis was reduced to 27.27% in the HELLS siRNA+TGFBR3 siRNA group from 32.82% in the HELLS siRNA group following treatment with 40 μM cisplatin (Figure 6G). These data confirmed that TGFBR3 is partially responsible for HELLS knockdown‐mediated sensitization to cisplatin in PC cells.

## 4 DISCUSSION

HELLS, a member of the SNF2 family of chromatin remodeling enzymes, was previously suggested to be overexpressed in various cancer types, such as colorectal cancer, HCC, nasopharyngeal carcinoma, and lung cancer.^16^, ^17^, ^18^, ^27^ For example, HCC‐upregulated HELLS is closely associated with cancer phenotypes, TP53 status, metastasis, and histological grade.^17^ The expression of HELLS is significantly elevated in the advanced clinical stage of nasopharyngeal carcinoma compared with that in the early stage.^16^ Consistent with these reports, we found that HELLS was highly expressed in PC and patients with higher expression showed higher grades of clinical stages and a poor prognosis. These data emphasized the clinicopathological significance of HELLS in PC and suggested its potential role in tumorigenesis in PC.

Epigenetic changes, including DNA methylation, chromatin remodeling, histone modification, and noncoding RNAs, are the key bioprocesses for tumorigenesis and tumor growth.^7^ Chromatin remodelers, such as HELLS, depend on the energy from ATP hydrolysis restructure of densely packed nucleosomes to improve chromatin accessibility.^28^ Dysregulation of chromatin remodeling disrupts the expression of tumor suppressor genes or otherwise stimulates the expression of oncogenes or oncogenic signaling pathways.^29^, ^30^, ^31^ An interesting study unveiled that HELLS interacts with DNMTs and HDACs to epigenetically silence target genes.^32^ Thus, apart from influencing chromatin accessibility, cross talk with DNA methylation regulators and histone deacetylases greatly complicate the role of HELLS in the epigenetic regulation of gene expression.^33^ A recent study demonstrated that the knockout of HELLS leads to global hypomethylation of the HCC genome; however, nevertheless no significant DNA methylation variation was observed in HELLS‐regulated genes.^17^ HELLS was reported to recruit histone methyltransferase G9a to repress fumarate hydrate, which is the key component of TCA.^16^ Furthermore, HELLS could reflow the nucleosome position and hinder NFR at the TSS of target genes, causing the suppression of several tumor suppressor genes in HCC.^17^ In our study, we found hundreds of genes with a difference in expression by more than twofold after HELLS knockdown in the three PC cell lines; among them, 23 genes differed among the cell lines, including several tumor suppressor genes, such as TGFBR3, BTG2, and DKK1. Functionally, the knockdown of HELLS impairs cell proliferation and reduces the ability of colony formation in vitro and tumor growth in vivo. We speculated that the overexpression of HELLS promotes PC growth by repressing several tumor suppressors.

In our analysis, TGFBR3 is one of the most significantly upregulated genes following HELLS knockdown in PC cells and TGFBR3 knockdown rescues HELLS knockdown‐induced growth arrest, indicating that TGFBR3 is an essential downstream mediator of HELLS in PC cells. TGFBR3 is the most abundant receptor of transforming growth factor‐β (TGF‐β) signaling that facilitates TGFBR2 binding with specific ligand and activates TGFBR1 kinase to phosphorylate canonical or noncanonical downstream mediators.^34^, ^35^ TGFBR3 frequently serves as a tumor suppressor in various cancer types, such as breast cancer, prostate cancer, and lung cancer.^36^, ^37^, ^38^ When the tumor progresses, the expression of TGFBR3 decreases and is well correlated with the poor prognosis of patients. Overexpression of TGFBR3 leads to cell proliferation arrest, deceased migration and invasion in vitro, and hinders angiogenesis and tumor growth in vivo.^39^ Notably, accumulating evidence has demonstrated that TGFBR3 is a tumor suppressor in PC. miR‐193a, a highly expressed microRNA in PC, suppresses TGFBR3‐mediated SMAD4 recruitment and blocks cell proliferation and metastasis upon radiation.^40^ Knockdown of TGFBR3 increased PC cell motility via soluble TGFBR3 but not depending on its cytoplasmic domain or coreceptor function.^41^ Although our work demonstrated that TGFBR3 functions downstream of HELLS, the precise mechanism underlying how TGFBR3 affects PC growth needs to be further elucidated.

A previous report showed that the ability of nonhomologous end joining is remarkably inhibited in HELLS‐deficient cells and its key components cause delayed accumulation at DNA damage sites, resulting in elevated DNA damage signals.^25^ Deletion of HELLS comprises nonhomologous end joining and leads to cell cycle arrest by increasing the early S population and increasing apoptosis following treatment with DNA damage agents.^25^ Furthermore, knockdown of HELLS was found to decrease the expression of numerous metabolic genes and inhibit ferroptosis, a newly recognized programed cell death in cancer.^42^ These lines of evidence indicate that HELLS is an active participant in the regulation of cell death besides its relatively elucidated role in cell growth. Our data verified that the downregulation of HELLS significantly improves DNA damage and apoptosis following cisplatin treatment. Mechanistically, the knockdown of HELLS desuppresses the expression of TGFBR3, which increases the activities of caspase 3, caspase 9, and Bax, followed by the induction of apoptosis through the mitochondrial pathway. The overexpression of TGFBR3 facilitates apoptosis through increasing the expression of proapoptotic proteins such as cleaved caspase 3, Bax, and Bcl‐2.^43^ Nevertheless, a report suggested that the ectopic expression of TGFBR3 largely abrogates hypoxia‐induced apoptosis by reversing the upregulation of Bax, Bcl‐2, and caspase 3 in cardiac fibroblasts.^44^ This discrepancy pinpoints the context‐dependent pattern of TGFBR3 in the regulation of apoptosis. Thus, a more detail investigation concerning the role of the HELLS/TGFBR3 axis in chemotherapy‐induced cell death should be performed.

In summary, our work firmly demonstrated an oncogenic role of HELLS, which promotes tumor growth and decreases the sensitivity to cisplatin in PC. We further unveiled the tumor suppressor TGFBR3 as a downstream target of HELLS, which is epigenetically silenced by HELLS. A thorough understanding of HELLS‐mediated epigenetic regulation of TGFBR3 will be needed that might involve a coordinated interplay among DNA methylation, histone modifications, and nucleosome structure.

## AUTHOR CONTRIBUTION

X. H. conducted the most assays; L. Y., K. W., Y. Z., and F. K., helped the assays; Q. L. provided the funding; J. H. designed the project and wrote the manuscript.

## Funding information

This work was supported by the Hunan Provincial Natural Science Foundation (2020JJ4790).

## Data Availability

We will provide original data when requested.
